# Transcriptional deciphering of the metabolic pathways associated with the bioactive ingredients of wolfberry species with different quality characteristics

**DOI:** 10.1186/s12864-023-09755-x

**Published:** 2023-11-02

**Authors:** Xuexia Liu, Rui Zheng, Yasmina Radani, Han Gao, Sijun Yue, Wenqiang Fan, Jianning Tang, Jing Shi, Jinzhong Zhu

**Affiliations:** 1 Key Laboratory of Ministry of Education for Protection and Utilization of Special Biological Resources in Western China, Key Laboratory of Modern Molecular Breeding for Dominant and Special Crops in Ningxia, College of Life Science, Ningxia University, Yinchuan, 750021 China; 2 College of Biology and the Environment, Nanjing Forestry University, Nanjing, 210037 China; 3 Ningxia Wolfberry Industry Development Center, Yinchuan, 750021 China; 4 Qixin Wolfberry Seedling Professional Cooperatives, Zhongning, 755100 China

**Keywords:** *Lycium barbarum* L., *Lycium chinense* Miller, Transcriptome, Differentially expressed genes, Bioactive ingredients

## Abstract

**Background:**

Wolfberry is rich in carotenoids, flavonoids, vitamins, alkaloids, betaines and other bioactive ingredients. For over 2,000 years, wolfberry has been used in China as a medicinal and edible plant resource. Nevertheless, the content of bioactive ingredients varies by cultivars, resulting in uneven quality across wolfberry cultivars and species*.* To date, research has revealed little about the underlying molecular mechanism of the metabolism of flavonoids, carotenoids, and other bioactive ingredients in wolfberry.

**Results:**

In this context, the transcriptomes of the *Lycium barbarum* L. cultivar ‘Ningqi No. 1’ and *Lycium chinense* Miller were compared during the fruit maturity stage using the Illumina NovaSeq 6000 sequencing platform, and subsequently, the changes of the gene expression profiles in two types of wolfberries were analysed. In total, 256,228,924 clean reads were obtained, and 8817 differentially expressed genes (DEGs) were identified, then assembled by Basic Local Alignment Search Tool (BLAST) similarity searches and annotated using Gene Ontology (GO), Clusters of Orthologous Groups of proteins (KOG), and the Kyoto Encyclopedia of Genes and Genomes (KEGG). By combining these transcriptome data with data from the PubMed database, 36 DEGs related to the metabolism of bioactive ingredients and implicated in the metabolic pathway of carotenoids, flavonoids, terpenoids, alkaloids, vitamins, etc., were identified. In addition, among the 9 differentially expressed transcription factors, *LbAPL*, *LbPHL11* and *LbKAN4* have raised concerns. The protein physicochemical properties, structure prediction and phylogenetic analysis indicated that *LbAPL* and *LbPHL11* may be good candidate genes involved in regulating the flavonoid metabolism pathway in wolfberry.

**Conclusions:**

This study provides preliminary evidence for the differences in bioactive ingredient content at the transcription level among different wolfberry species, as well as a research and theoretical basis for the screening, cloning and functional analysis of key genes involved in the metabolism of bioactive ingredients in wolfberry.

## Introduction

Wolfberry, a solanaceous perennial shrub, is widely used as a functional food and herbal medicine for its biological and pharmacological activity [1, 2]. It has been reported to play a significant role in lowering blood sugar and serum lipids, in addition to having antiaging, immunomodulatory, and antitumour properties, together with other functions [3, 4], mainly due to its various bioactive ingredients, such as *Lycium barbarum* polysaccharides (LBPs), flavonoids, carotenoids, betaines, and alkaloids [5]. LBPs, for example, possess antiaging, antiapoptotic, and anti-inflammatory properties [6, 7]. Carotenoids (β-carotene and zeaxanthin) have the potential to induce cancer cell apoptosis and prevent heart disease and stroke [8]. Consequently, there has recently been considerable interest in the bioactive ingredients found in wolfberry.

**Fig. 1:**
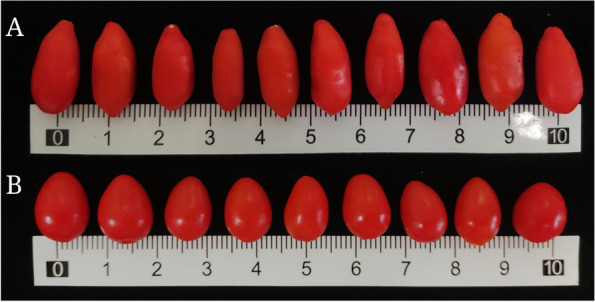
Fresh fruit appearance of ‘Ningqi No.1’ (**A**) and *L. chinense* (**B**)

It is well known that differences in wolfberry species lead to variations in their bioactive ingredient content. The *Lycium* genus includes approximately 100 species worldwide, the majority of which are found in North and South America, with 7 species and 2 varieties found in Northwest and North China [9]. *Lycium barbarum* L. and *Lycium chinense* Miller, two closely related species, are currently the best-selling wolfberries, with nearly 90% of all commercially available wolfberries belonging to the former, which is the only wolfberry species listed in the *Chinese Pharmacopoeia.* As a *L. barbarum* cultivar, ‘Ningqi No. 1’ is the most widely cultivated variety in China because of its superior quality, high yield, strong adaptability and other characteristics. In addition, the fruits of ‘Ningqi No. 1’ possess the unique morphological features of having fruits that are fusiform or oblong in shape (Fig. 1) and this cultivar is commonly grown in northwestern China’s Ningxia Hui Autonomous Region and Xinjiang Uyghur Autonomous Region. Unlike the fruits of ‘Ningqi No. 1’, *L. chinense* fruits are elliptical in shape (Fig. 1) and can be found in Northwest China and other warm and subtropical countries, such as Korea, Japan, and some European countries [3]. Additionally, there is a difference between ‘Ningqi No. 1’ and *L. chinense* in the flavour of the fruit, with the fruit of the former tasting only sweet, whereas the fruit of the latter tastes sweet with some bitterness. Remarkably, current findings indicate that the polysaccharide content of *L. chinense* fruit is approximately 12.36 mg/g, which is three times that of ‘Ningqi No. 1’ fruit. However, the content of betaine and vitamin C in the fruit of *L. chinense* are 4.89 mg/g and 1.38 mg/g, respectively, which are lower by approximately 4.68% and 25.41%, respectively, than the content of these bioactive ingredients in the fruit of ‘Ningqi No. 1’ [10]. Clearly, the fruits of ‘Ningqi No. 1’ and *L. chinense* differ in appearance and internal quality. The essence of such uneven fruit quality is attributed to differences in gene expression and to genetic and environmental factors.

Transcriptomics is a technique for studying gene expression and transcriptional regulation at a large scale, and it is an effective research tool for studying the relationship between cell phenotype and gene function. RNA-Seq has been utilized to discover and identify functional candidate genes involved in plant growth, development and secondary metabolite biosynthesis, as it is an affordable and trustworthy method for transcriptome analysis. For example, Wen et al. [11] found some unigenes that are closely related to chlorogenic acid content, revealing the potential mechanism of chlorogenic acid biosynthesis through RNA-seq along with de novo transcriptome analysis in Korla fragrant pear grown in the Xinjiang region of China. Similarly, a comparative transcriptome analysis of *Strombosiopsis tetrandra* roots and leaves was performed, revealing potential genes that regulate the biosynthesis of isoquinoline alkaloids [12]. In *L. barbarum*, numerous enzyme genes associated with carotenoid biosynthesis [3] and phenylpropanoid biosynthesis [13] were identified through transcriptome analysis. Previous research on *L. chinense* has primarily concentrated on basic biological research such as nutritional component extraction and separation [6], pharmacology and medicinal function [14, 15], and investigation of candidate genes participated in the biosynthesis of vital secondary metabolites [3, 13]. However, there are few studies to reveal the variations in bioactive ingredient content at the molecular level among multiple species of wolfberry. More importantly, this information about gene expression is of great significance for studying the molecular mechanisms of genetic evolution as well as further improving wolfberry species. Therefore, this study identified several differentially expressed genes associated with the metabolism of bioactive ingredients within the fruits of ‘Ningqi No. 1’ and *L. chinense* through RNA-Seq. The ultimate goal was to elucidate the molecular basis for differences in fruit bioactive components between these two types of wolfberries and to provide basic data and candidate genes for improving wolfberry fruit quality*.*

## Results

### RNA-seq analysis

**Fig. 2:**
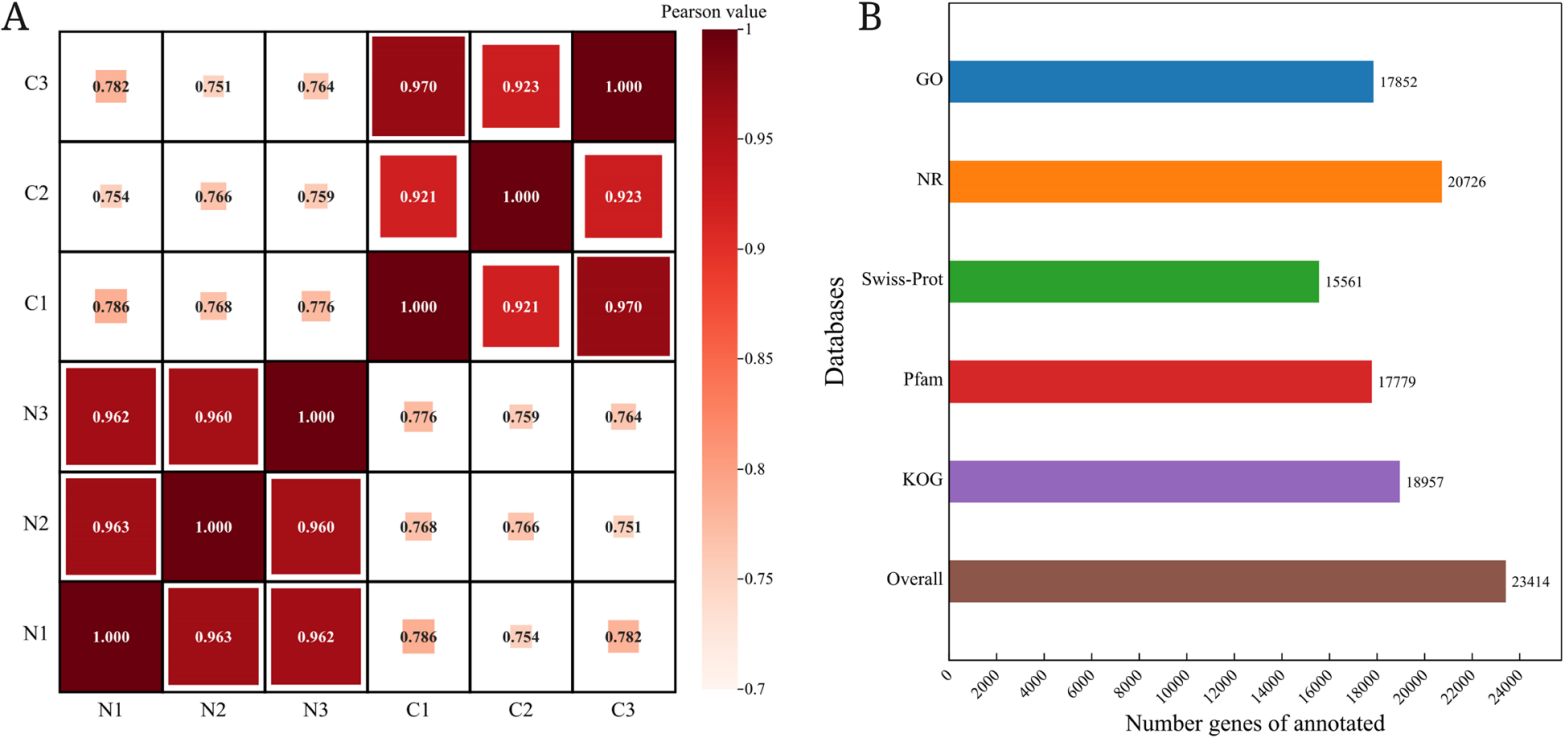
**A** Individual correlation analyses between 6 transcriptome samples of ‘Ningqi No. 1’ and *L. chinense*. **B** Gene annotations of ‘Ningqi No. 1’ and *L. chinense*

To provide a detailed summary of the transcriptome and gene expression differences between ‘Ningqi No. 1’ and *L. chinense* fruits, 6 cDNA samples from each of the two types (with each sample having three biological replicates) were prepared and sequenced using Illumina NovaSeq 6000. Following a thorough quality assessment and data screening, 256,228,924 clean reads were obtained. The Q20 (nucleotide ratio with a quality value greater than 20 in the reads) and Q30 (nucleotide ratio with a quality value greater than 30 in the reads) percentages of each sample were greater than 97.28% and 92.30%, respectively. The GC content of each sample (percentage of GC nucleotide content in high-quality reads) was 41.86% ~ 42.30%. The clean reads were then aligned with the designated *Lycium barbarum* reference genome to obtain mapped data for subsequent transcript assembly, expression computation, and so on. Consequently, the mapping rate ranged from 84.80% to 95.45% (Table 1). Considering that the correlation of biological duplication is very important for analysing transcriptome sequencing data, Pearson correlation analysis was performed on the fruit samples of ‘Ningqi No. 1’ and *L. chinense* during the maturity stage. The correlation coefficient was 0.7 < *R*^2^ < 1 (Fig. 2A), manifesting that the 6 samples presented a relatively high degree of homogeneity of genes within each sample. Overall, these findings showed that RNA-seq produced the high-quality data and reflected a high assembly integrity, which demonstrated that the experimental data could be subsequently analysed.

**Table 1:** Statistics of sequencing data (N1, N2 and N3 are three replicates of the ‘Ningqi No.1’ fruits. C1, C2 and C3 are three replicates of the *L. chinense* fruits.)

Sample	Clean reads	Clean bases(G)	Q20 (%)	Q30 (%)	GC content (%)	Mapping rate (%)
N1	42,117,386	6.32	97.77	93.58	42.30	95.45%
N2	43,947,310	6.59	97.43	92.68	42.28	95.38%
N3	45,400,236	6.81	97.56	93.08	41.86	94.91%
C1	39,611,032	5.94	97.28	92.30	41.92	85.13%
C2	40,908,772	6.14	97.33	92.43	41.91	84.80%
C3	44,244,188	6.64	97.38	92.52	42.07	85.19%

### Functional annotation

A total of 23,414 expressed genes had the most remarkable BLAST matches to known proteins in the six public databases, KEGG, GO, NR, Swiss-Prot, Pfam, and KOG (Fig. 2B). Among the six databases, NR had the most annotated genes with 88.52% (20,726), while the Swiss-Prot database had the lowest rate, at 66.46% (15,561).

### DEG analysis of ‘Ningqi No. 1’ and *L. chinense*

**Fig. 3:**
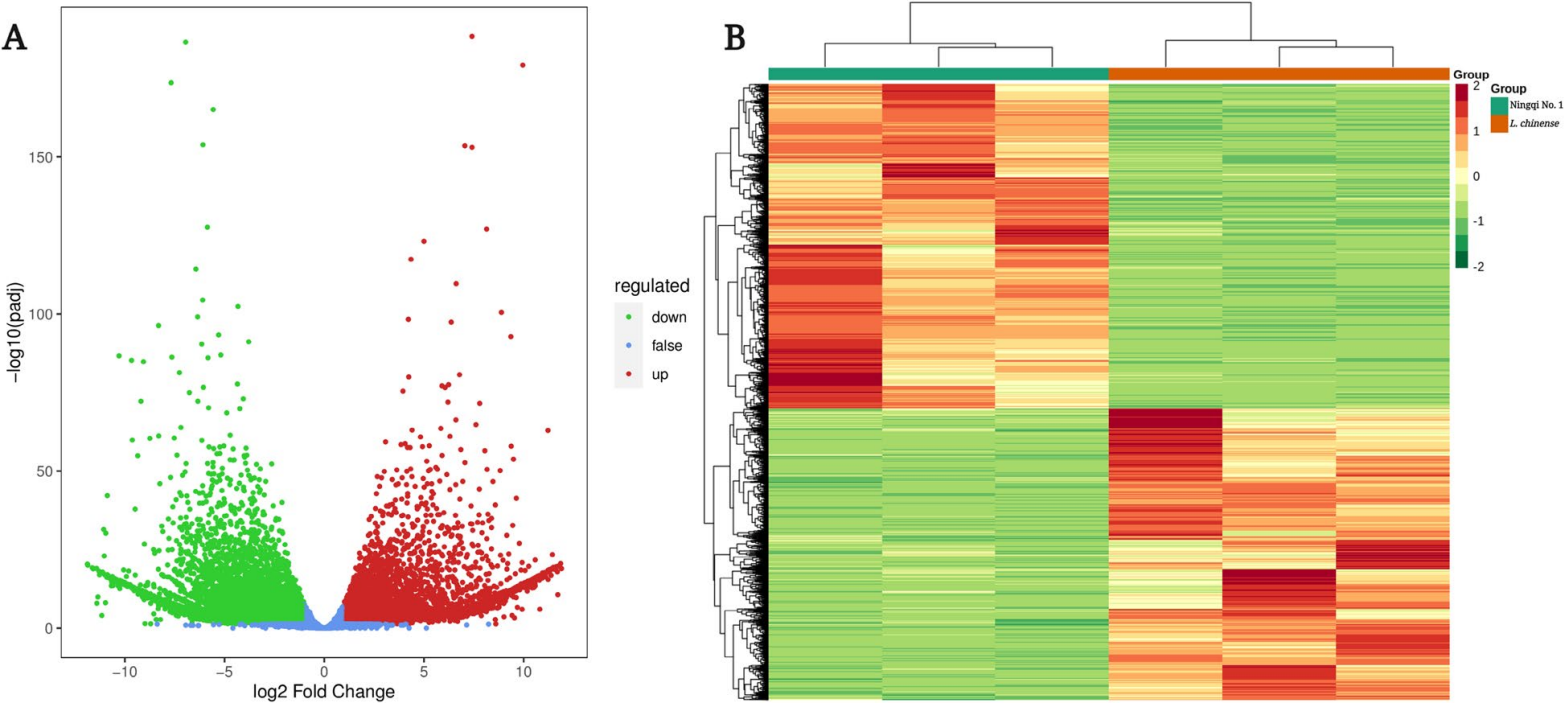
Volcano map (**A**) and hierarchical cluster analysis (**B**) of DEGs

To identify DEGs, a comparative transcriptome analysis of ‘Ningqi No. 1’ and *L. chinense* fruits was performed using ‘Ningqi No. 1’ fruits as controls. A total of 8817 DEGs were found, with 4036 genes upregulated and 4181 genes downregulated (Fig. 3), indicating that the gene expression patterns of ‘Ningqi No. 1’ and *L. chinense* differed greatly.

**Fig. 4:**
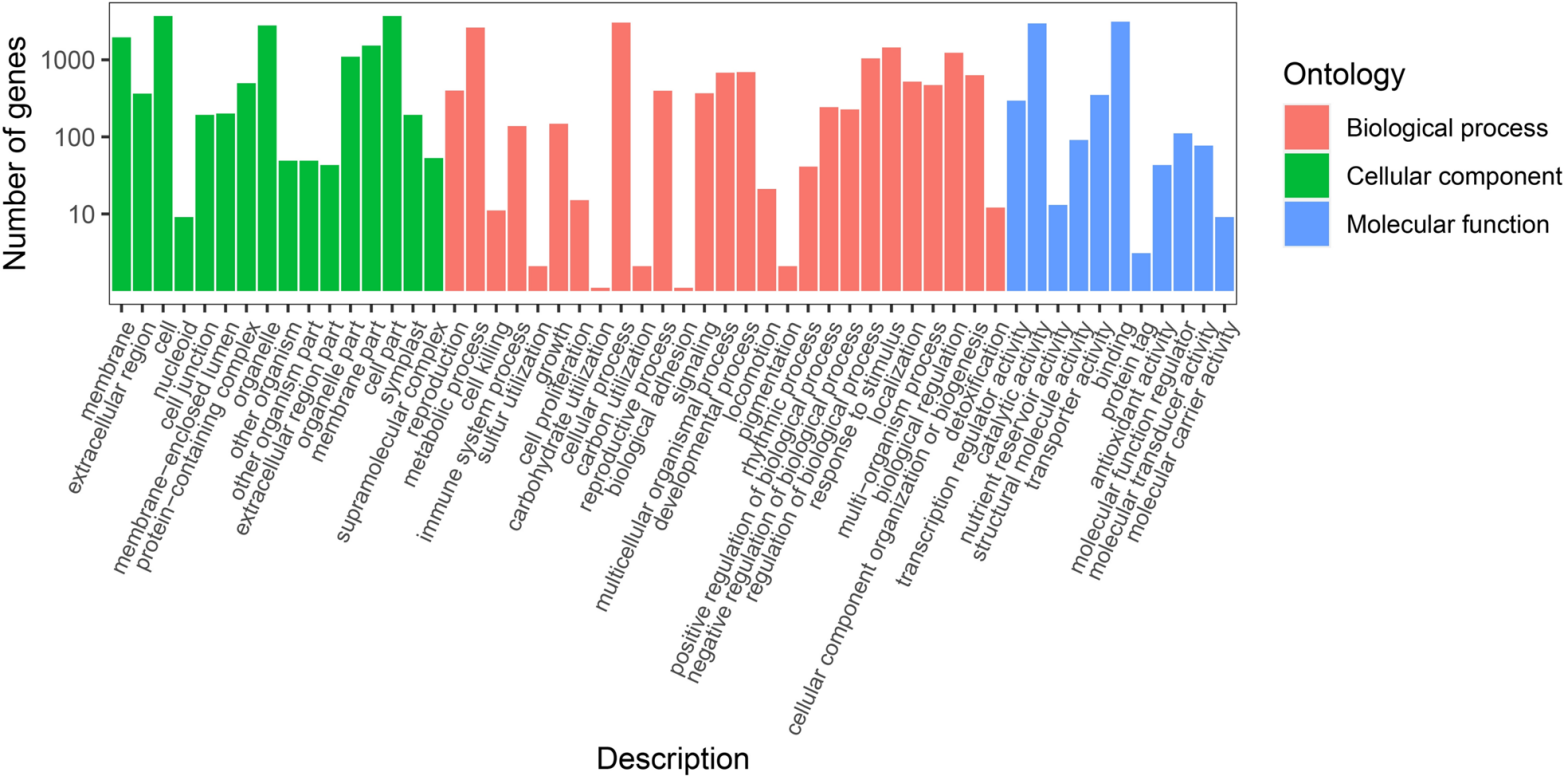
GO classification of DEGs

**Fig. 5:**
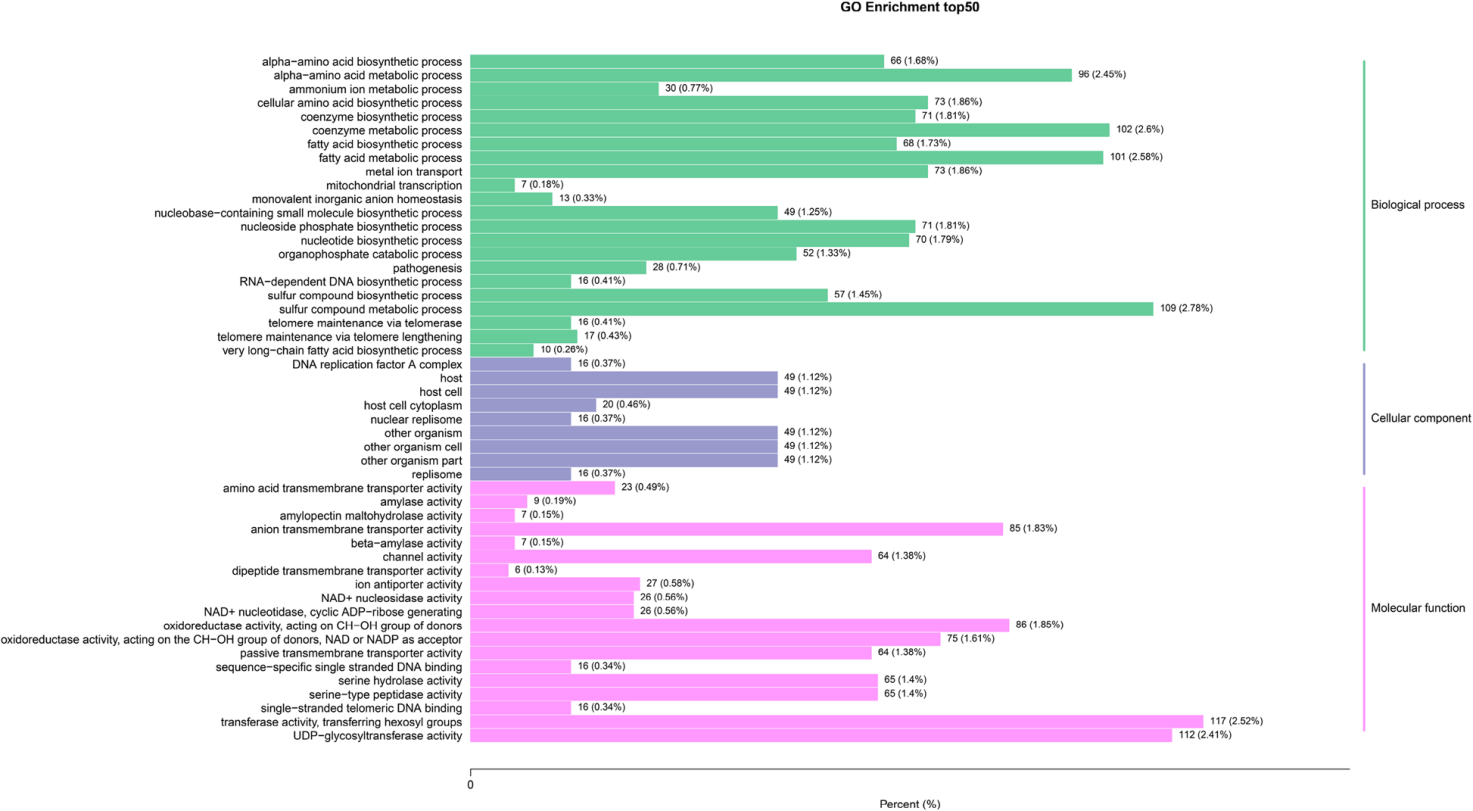
GO enrichment of DEGs

As the world’s largest source of classification information and gene function, the GO database is grouped into three categories: Cellular components (CC), Biological processes (BP) and Molecular function (MF). To determine the functional distribution of the genes, GO annotation was used to classify the DEGs from ‘Ningqi No. 1’ and *L. chinense*, with the results revealing that these genes were divided into three predominant functional categories, CC, BP and MF, with 54 subcategories (Fig. 4). Specifically, the CC category was divided into 16 subcategories, with ‘cell’ and ‘cell part’ containing the most DEGs, at 3699 (41.95%), followed by ‘organelle’ with 2788 (31.62%) DEGs. The BP category was further subdivided into 27 subcategories, with ‘cellular process’ and ‘metabolic process’ being the two main subcategories, including 3040 (34.48%) and 2619 (29.70%) DEGs. The MF category was mapped into 11 GO terms, with the most plentiful subcategories being ‘binding’ and ‘catalytic activity’, which had 3127 (35.47%) and 2965 (33.63%) DEGs, respectively. The functional classification of all the DEGs could be crudely understood after the GO annotation. Furthermore, a GO enrichment analysis was performed to investigate the distribution of DEGs to clarify differences in gene function among samples. The 50 GO terms with a significant enrichment of DEGs showed that those belonging to BP were the most numerous, with a total of 22. The highest proportion of the 50 GO terms was accounted for by the sulfur compound metabolic process (2.78%), transferase activity, transferring hexosyl groups (2.52%) and UDP-glycosyltransferase activity (2.41%) (Fig. 5).

**Fig. 6:**
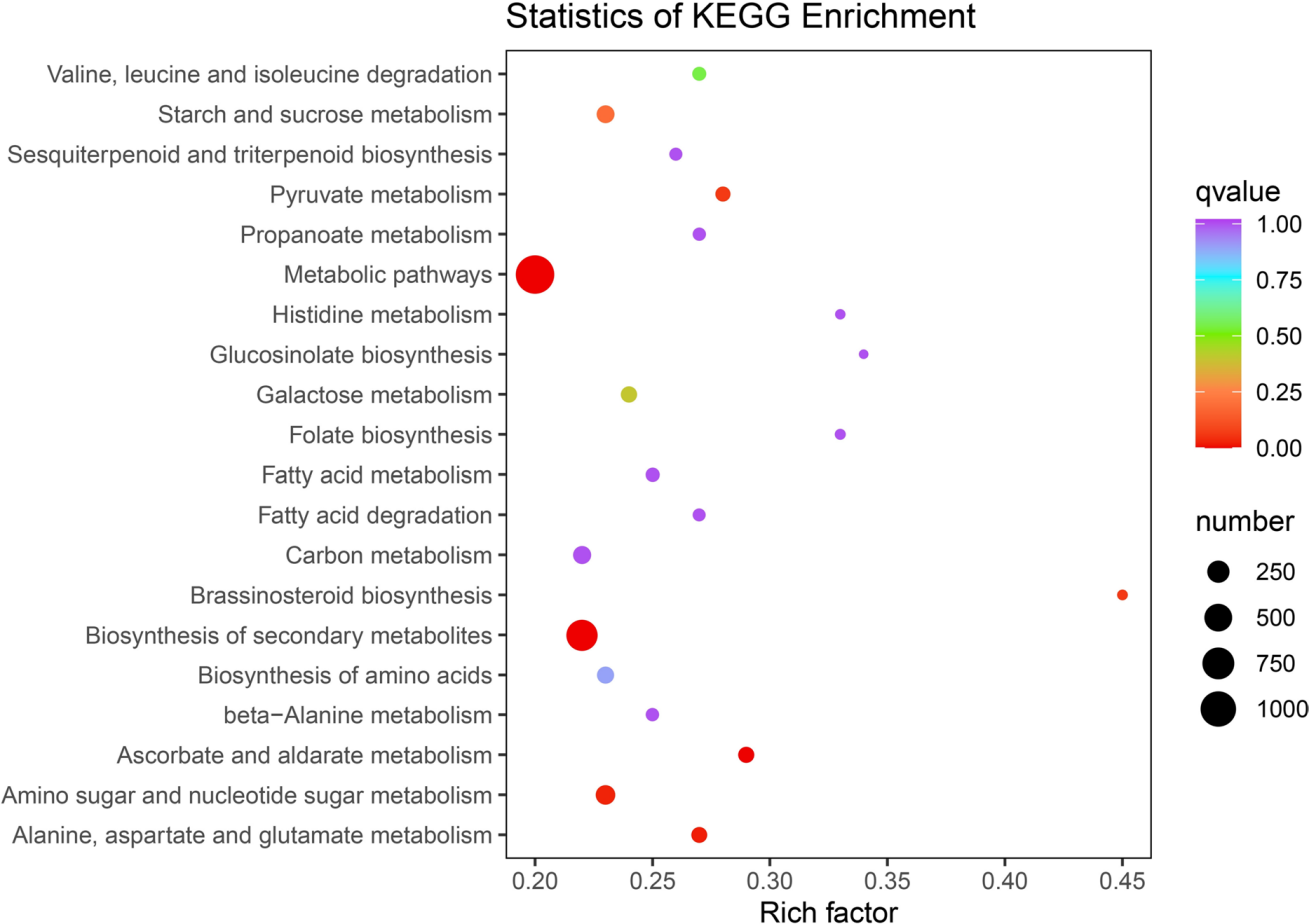
KEGG analysis of DEGs

The KEGG tool can organically combine genomic with functional information and systematically analyse the metabolic pathways of each gene product in the cell, so as to more comprehensively understand the functions of these gene products. Therefore, KEGG annotation and enrichment analysis of the DEGs in ‘Ningqi No. 1’ and *L. chinense* was performed. The results demonstrated that a total of 139 pathways were annotated, with the most significant enrichment in ‘Metabolic pathways’, ‘Biosynthesis of secondary metabolites’, ‘Brassinosteroid biosynthesis’, ‘Pyruvate metabolism’, ‘Ascorbate and aldarate metabolism’, ‘Amino sugar and nucleotide sugar metabolism’ and ‘Alanine, aspartate and glutamate metabolism’. Notably, the 20 pathways that were significantly enriched included ‘sesquiterpenoid and triterpenoid biosynthesis’, ‘folate biosynthesis’, and ‘ascorbate and aldehyde metabolism’. The DEGs enriched in these metabolic pathways were related to the metabolism of bioactive ingredients, implying that there might be many DEGs correlated with the difference in bioactive component accumulation in fruits (Fig. 6).

### Analysis of DEGs related to the metabolism of bioactive ingredients

**Fig. 7:**
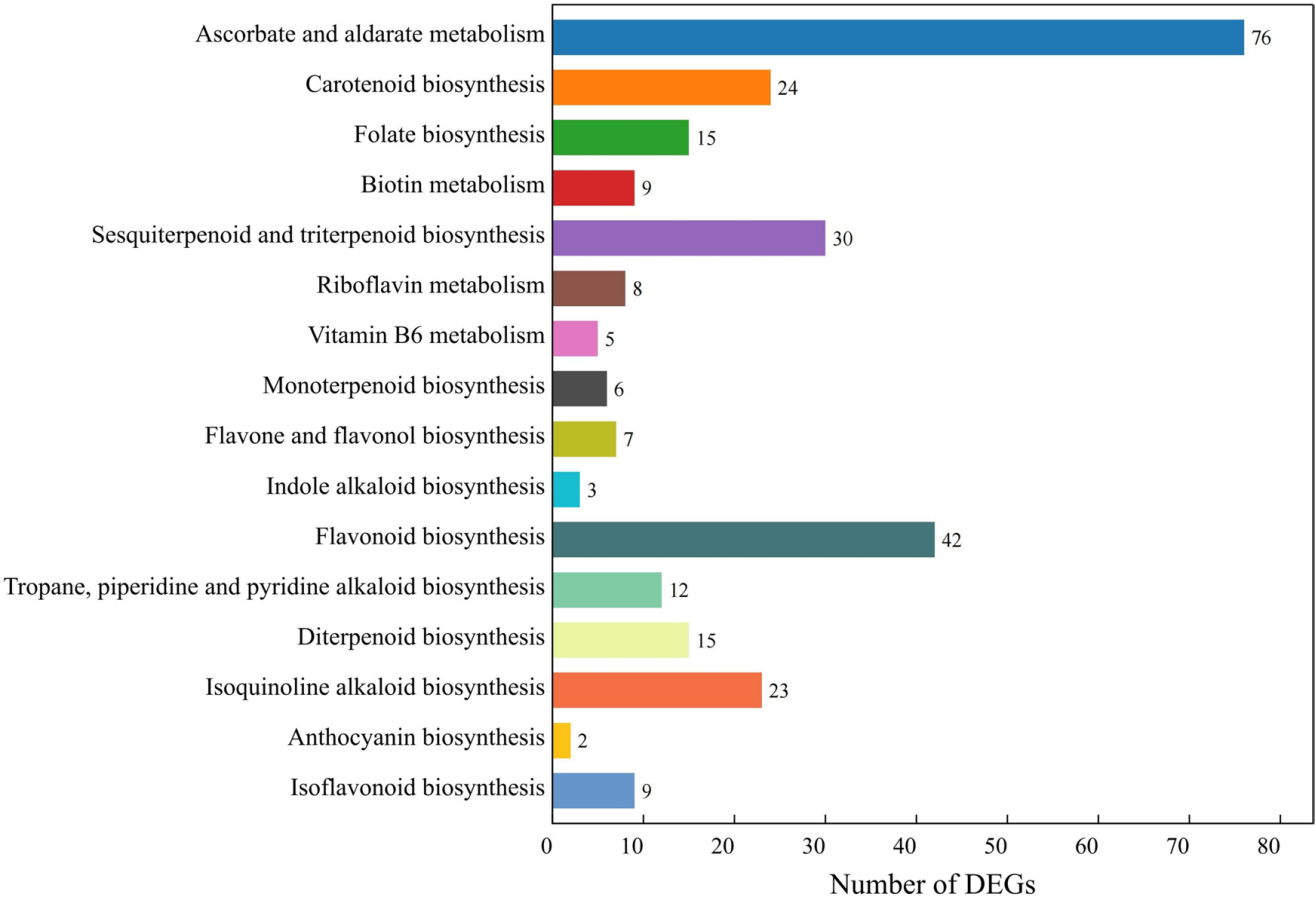
The KEGG pathways related to bioactive ingredients metabolism

The DEGs can each be located in 139 specific metabolic pathway branches, including 16 pathways related to bioactive ingredient metabolism, in the KEGG database [16–18] (Fig. 7). The metabolic pathway for ascorbate and aldarate had the most annotated DEGs, with 76 DEGs. Additionally, the flavonoid metabolic pathway, which included ‘flavone and flavonol biosynthesis’, ‘flavonoid biosynthesis’, ‘anthocyanin biosynthesis’ and ‘isoflavonoid biosynthesis’, was annotated with a total of 60 DEGs. The terpenoid metabolic pathway, which includes ‘sesquiterpenoid and triterpenoid biosynthesis’, ‘monoterpenoid biosynthesis’ and ‘diterpenoid biosynthesis’, was also highly annotated, with 51 DEGs, indicating that there may be many good candidate genes associated with the metabolism of bioactive ingredients found in fruits, such as flavonoids and terpenoids.

**Fig. 8:**
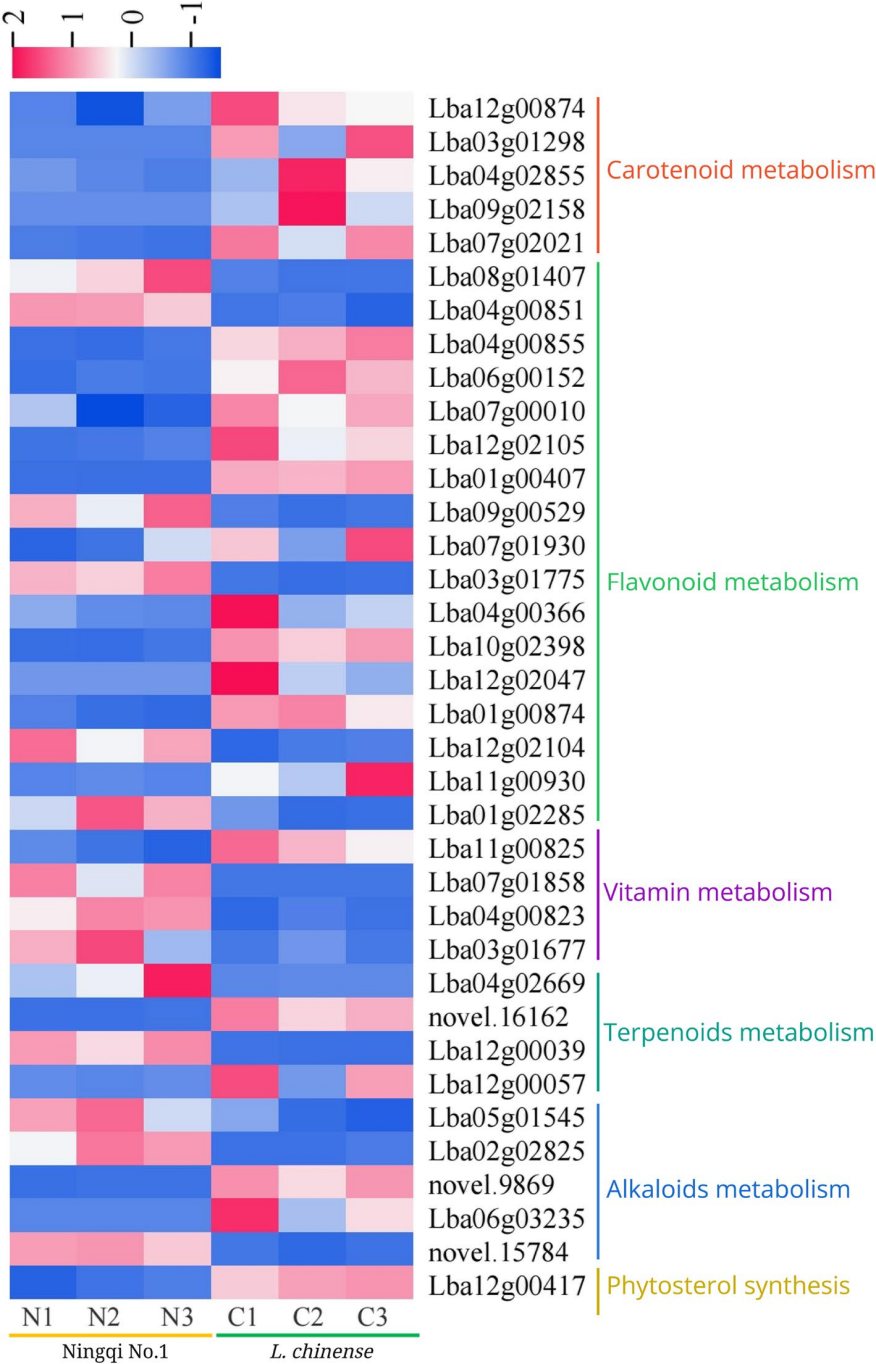
Heatmap of 36 DEGs related to metabolism of active ingredients

According to the KEGG pathway annotation, combined with the information from the NR and PubMed databases, 36 DEGs associated with the bioactive ingredient metabolism were identified (Table 2), and then whose expression characteristics in ‘Ningqi No. 1’ and *L. chinense* were determined by the fragments per kilobase of transcript per million mapped reads (FPKM) values of the transcriptome data (Fig. 8). *L. chinense* had 21 genes with high expression and 15 genes with low expression when compared to ‘Ningqi No. 1’. These DEGs included five key carotenoid biosynthesis enzyme genes that separately encode phytoene synthase 2, phytoene desaturase, lycopene epsilon-cyclase, carotenoid cleavage dioxygenase 4, and 15-cis-zeta-carotene isomerase. It is also worth noting that these DEGs were all upregulated in *L. chinense*. In addition, there were 17 genes encode 8 enzymes associated with the flavonoid biosynthesis pathway, namely, phenylalanine ammonia-lyase, 4-coumarate–CoA ligase, anthocyanidin 3-O-glucosyltransferase 5-like, chalcone synthase, anthocyanidin synthase, anthocyanidin reductase-like, dihydvroflavonol-4-reductase and flavanone 3-hydroxylase-like. Moreover, four enzyme genes involved in vitamin metabolism, four enzyme genes related to terpenoid metabolism, 5 enzyme genes associated with alkaloid metabolism and 1 enzyme gene involved in phytosterol synthesis were identified. Notably, the vast majority of enzyme genes involved in the metabolism of vitamins, terpenoids, and alkaloids were found to be downregulated in *L. chinense*.

**Table 2:** DEGs related to bioactive ingredients metabolism of ‘Ningqi No.1’ and *L. chinense*

Gene Code	NR Annotation	Relative expression	Gene function
Lba12g00874	phytoene synthase 2	up	Carotenoid metabolism
Lba03g01298	phytoene desaturase	up	
Lba04g02855	lycopene epsilon-cyclase	up	
Lba09g02158	putative carotenoid cleavage dioxygenase 4	up	
Lba07g02021	15-cis-zeta-carotene isomerase	up	
Lba08g01407	phenylalanine ammonia-lyase	down	Flavonoid metabolism
Lba04g00851	4-coumarate–CoA ligase-like 6 isoform X1	down	
Lba04g00855	4-coumarate–CoA ligase-like 6 isoform X1	up	
Lba06g00152	4-coumarate–CoA ligase-like 7	up	
Lba07g00010	4-coumarate–CoA ligase 1	up	
Lba12g02105	anthocyanidin 3-O-glucosyltransferase 5-like	up	
Lba01g00407	anthocyanidin 3-O-glucosyltransferase 5-like	up	
Lba09g00529	chalcone synthase, partial	down	
Lba07g01930	chalcone synthase 2	up	
Lba03g01775	4-coumarate–CoA ligase 2	down	
Lba04g00366	anthocyanidin synthase	up	
Lba10g02398	anthocyanidin synthase-like	up	
Lba12g02047	dihydvroflavonol-4-reductase	up	
Lba01g00874	dihydroflavonol-4-reductase-like	up	
Lba12g02104	anthocyanidin 3-O-glucosyltransferase 5-like	down	
Lba11g00930	flavanone 3-hydroxylase-like	up	
Lba01g02285	flavanone 3-hydroxylase-like	down	
Lba11g00825	riboflavin synthase	up	Vitamin metabolism
Lba07g01858	lumazine synthase 1	down	
Lba04g00823	gamma-tocopherol methyltransferase	down	
Lba03g01677	pyridoxal reductase, chloroplastic-like	down	
Lba04g02669	beta-amyrin synthase	down	Terpenoids metabolism
novel.16162	beta-amyrin synthase-like	up	
Lba12g00039	dammarenediol II synthase-like	down	
Lba12g00057	dammarenediol II synthase-like	down	
Lba05g01545	vinorine synthase-like	down	Alkaloids metabolism
Lba02g02825	vinorine synthase-like	down	
novel.9869	(S)-N-methylcoclaurine 3'-hydroxylase isozyme 1-like	up	
Lba06g03235	polyneuridine-aldehyde esterase-like	up	
novel.15784	berberine bridge enzyme-like 8	down	
Lba12g00417	delta(7)-sterol-C5(6)-desaturase	up	Phytosterol synthesis

### Analysis of transcription factors related to bioactive ingredient metabolism

Transcription factors can ensure the expression of target genes at a specific time and in a particular space, and they are important elements of networks that regulate metabolism, development, response to biotic and abiotic stresses, and so on. By analysing the transcriptome data of ‘Ningqi No. 1’ and *L. chinense*, this study hopes to find the transcription factors that regulate the metabolism of active components in wolfberry and provide fundamental data for understanding their mechanism of action. As a result, among the 9 transcription factors that were differentially expressed, the MYB family transcription factor APL (LbAPL), PHL11 isoform X2 (LbPHL11) and transcription factor KAN4 (LbKAN4) were identified. They were downregulated in *L. chinense* compared to ‘Ningqi No. 1’.

#### Prediction of the physicochemical properties and structures of the transcription factors LbAPL, LbPHL11 and LbKAN4

The amino acid sequences of LbAPL, LbPHL11 and LbKAN4 were analysed by the NCBI (https://www.ncbi.nlm.nih.gov/) BLAST tool. The results showed that the three proteins all shared the SANT superfamily conserved domain, which belongs to the MYB protein superfamily.

ProtParam tool (https://web.expasy.org/protparam/) was applied to analyse the physicochemical properties of the LbAPL, LbPHL11 and LbKAN4 proteins. The findings revealed that the amino acid numbers of these transcription factors were 238, 280 and 401, the molecular weights were 26,558.29 Da, 31,415.25 Da and 45,287.20 Da, and the theoretical isoelectric points (pIs) were 9.37, 6.11 and 9.16, respectively. The instability indexes were all above 40, manifesting that they were unstable proteins. These transcription factors were hydrophilic proteins because of their grand average of hydropathicity (GRAVY) less than 0 (Table 3). The subcellular localization prediction results showed that the transcription factors LbAPL, LbPHL11 and LbKAN4 were in the nucleus.

**Table 3:** Physical and chemical characteristics of *Lycium barbarum* MYB transcription factor family proteins

Gene Code	Lba05g02492	Lba04g00863	Lba01g01402
Gene name	*LbAPL*	*LbPHL11*	*LbKAN4*
Number of amino acids	238	280	401
Molecular weight (Da)	26,558.29	31,415.25	45,287.20
Theoretical pI	9.37	6.11	9.16
Instability index	45.66	50.17	51.57
Aliphatic index	81.13	74.61	59.88
Grand average of hydropathicity (GRAVY)	-0.566	-0.709	-0.898

**Fig. 9:**
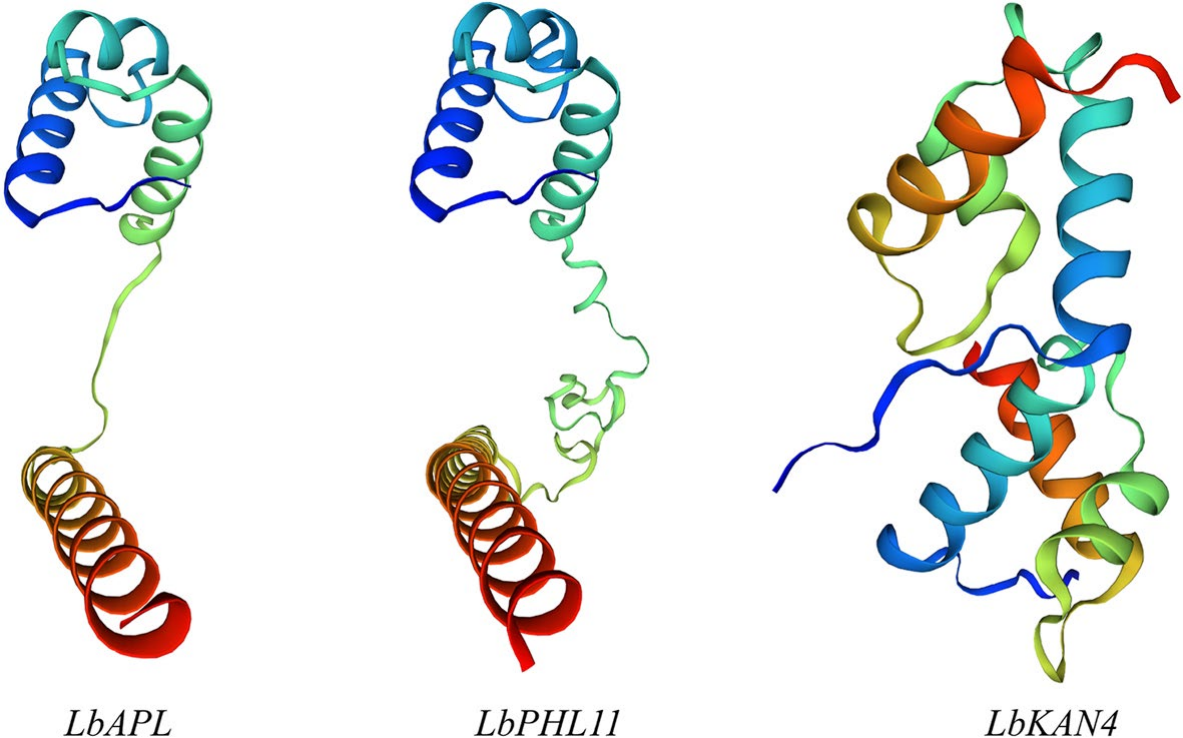
Tertiary structure prediction of *Lycium barbarum* MYB transcription factor family proteins

Based on their amino acid sequences, the secondary and tertiary structures of the LbAPL, LbPHL11 and LbKAN4 proteins were each predicted using Self-Optimized Prediction Method with Alignment (SOPMA) (https://npsa-prabi.ibcp.fr/cgi-bin/npsa_automat.pl?page=npsa_sopma.html) and SWISS-MODEL (https://swissmodel.expasy.org/) software in the ExPASy database. The results indicated that the alpha helix and random coil were the main components of these proteins’ secondary structure and that these components formed the spatial structure of these MYB proteins. Among the proteins, the LbAPL and LbPHL11 proteins showed high similarity in their tertiary structures (Fig. 9).

#### Phylogenetic analysis of LbAPL, LbPHL11 and LbKAN4 proteins

**Fig. 10:**
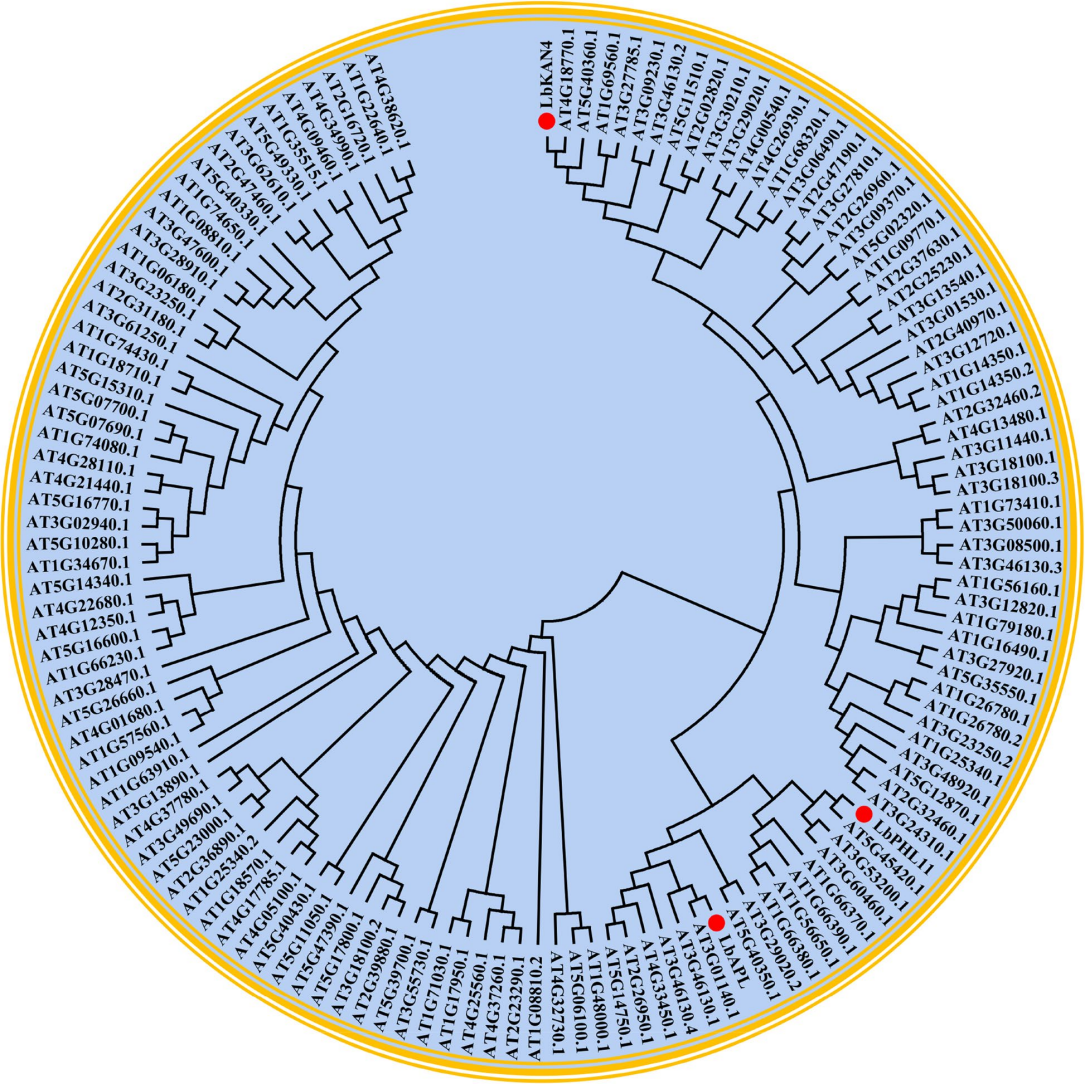
The phylogenetic tree of LbAPL, LbPHL11 and LbKAN4 transcription factors of *Lycium barbarum* and other MYB proteins involved in flavonoid metabolism from different species

Several MYB transcription factors participate in the regulation of flavonoid biosynthesis [19, 20]. MEGA 5.05 software was used in this study to construct a phylogenetic tree between the LbAPL, LbPHL11, and LbKAN4 proteins and the amino acid sequences of the *Arabidopsis thaliana* MYB family proteins (Fig. 10). The results showed that LbAPL had close ortholog with *Arabidopsis thaliana* MYB106 (at3g01140.1) and MYB48 (at3g46130.1). LbPHL11 had close ortholog with MYB27 (at3g53200.1) and MYB125 (at3g60460.1). LbKAN4 had close ortholog with MYB98 (at4g18770.1) and MYB115 (at5g40360.1). Therefore, the transcription factors LbAPL, LbPHL11 and LbKAN4 may have the same function as the *Arabidopsis thaliana* MYB protein from the same branch. In addition, it has been reported that *MYB27* is the repressor of the anthocyanin biosynthesis pathway, acting as a member of the MBW complex and repressing transcription via its C-terminal EAR motif [21]. *MYB48* regulates flavonol biosynthesis mainly in cotyledons [22]. Obviously, *MYB27* and *MYB48* were closely connected with the synthesis of flavonoids. Therefore, it could be hypothesized that *LbAPL* and *LbPHL11* might perform similar function in the regulation of flavonoid biosynthesis in wolfberry.

#### Analysis of *LbAPL*, *LbPHL11* and *LbKAN4* gene expression patterns

**Fig. 11:**
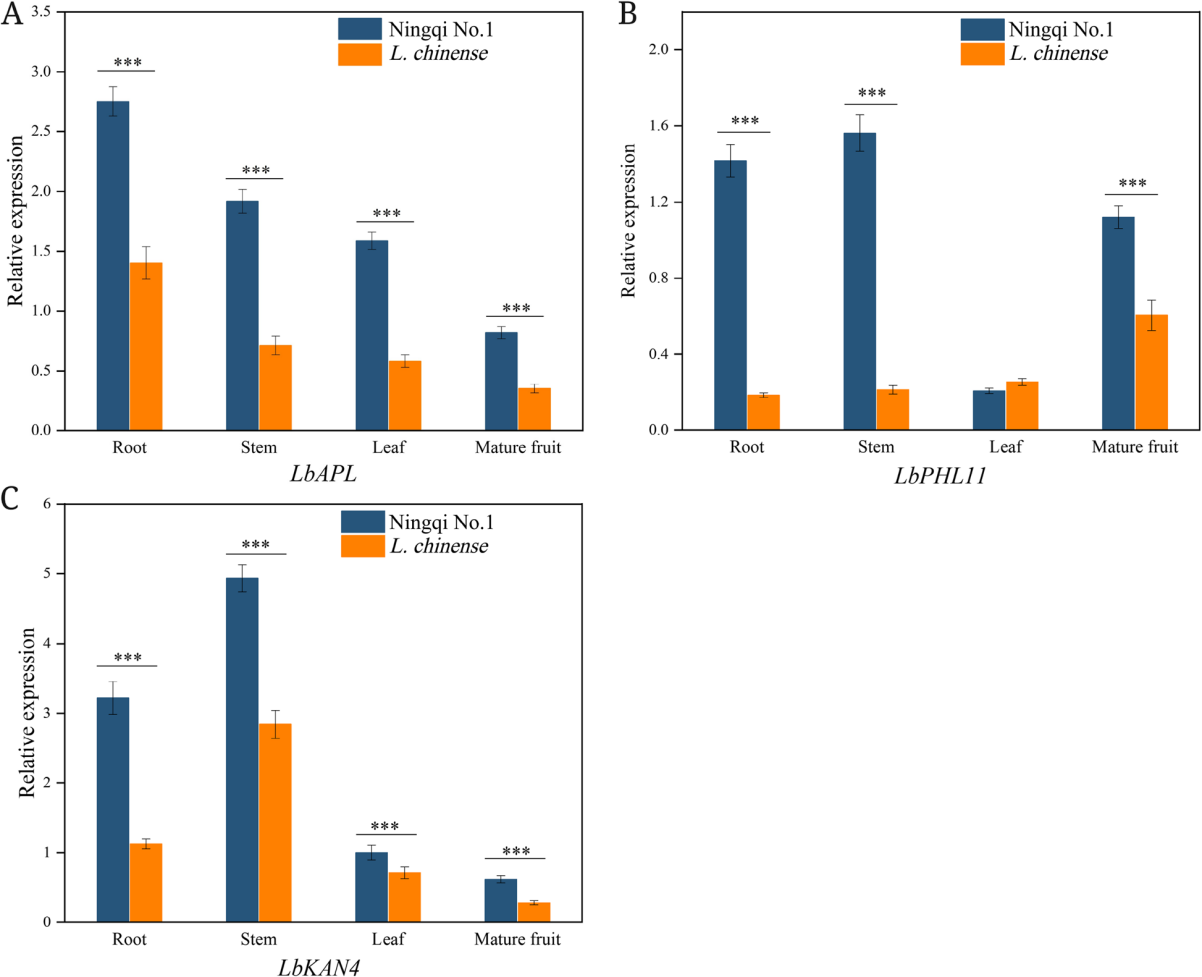
Relative expression levels of three transcription factors in different tissues of ‘Ningqi No.1’ and *L. chinense*. **A** *LbAPL*. **B** *LbPHL11. ***C** *LbKAN4*. Data are means (± SD) of three biological replicates. Asterisks ‘*’ represent statistical differences in the same index between different species, with *p* < 0.05 being a significant difference (*p < 0.05, **p < 0.01, ***p < 0.001)

The expression of the *LbAPL*, *LbPHL11* and *LbKAN4* genes in the roots, stems, leaves and mature fruits of ‘Ningqi No. 1’ and *L. chinense* was analysed through RT‒qPCR. The results indicated that the level of *LbAPL* expression gradually decreased in the roots, stems, leaves and mature fruits of ‘Ningqi No. 1’ and *L. chinense* (Fig. 11A). In ‘Ningqi No. 1’, *LbPHL11* expression was abundant in stems, roots and mature fruit but low in leaves. However, the level of *LbPHL11* expression in mature fruits was higher than that in other tissues in *L. chinense* (Fig. 11B). *LbKAN4* expression was highest in the stems of both ‘Ningqi No. 1’ and *L. chinense*, followed by the roots, and was lowest in the mature fruits (Fig. 11C). Except for *LbPHL11* in the leaves of ‘Ningqi No. 1’, all tissues of ‘Ningqi No. 1’ expressed higher levels of *LbAPL*, *LbKAN4* and *LbPHL11* than *L. chinense*.

### Analysis of carotenoid, flavonoid, and isoflavone content in the fruit maturity stage of the two wolfberry types

**Fig. 12:**
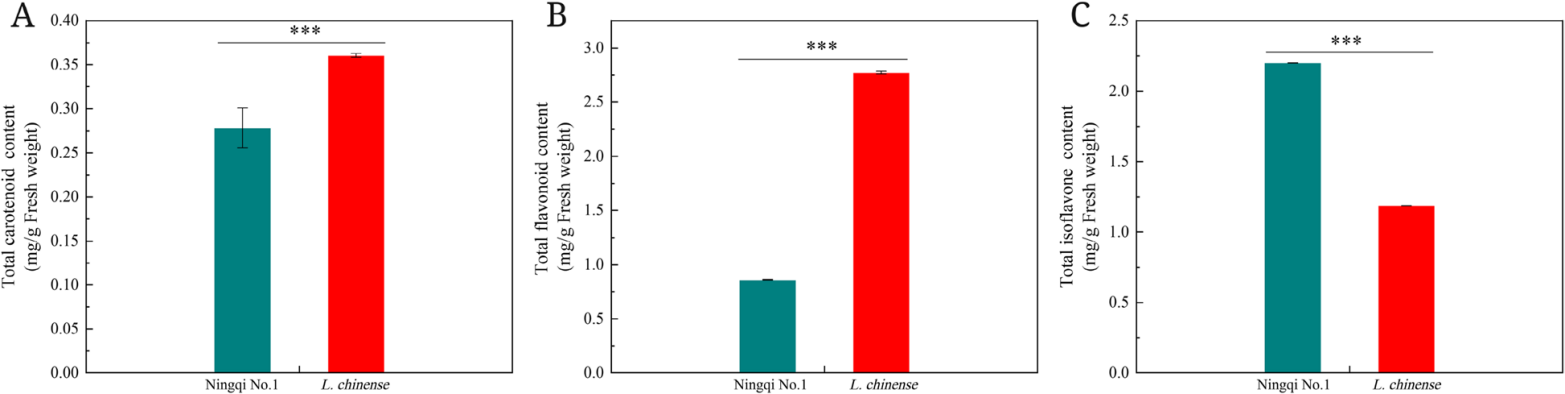
**A** Total carotenoid content, **B** Total flavonoid content, **C** Total isoflavone content. The bars represent mean (*n* = 3), and the error bars indicate standard deviation (SD). The asterisks (***) reveal the high significant difference at *p* < 0.001

We further quantitatively profiled the carotenoids, flavonoids and isoflavones between the two types of wolfberries based on the higher number of DEGs involved in carotenoid and flavonoid biosynthesis. The results suggested that the total carotenoid content detected in ‘Ningqi No. 1’ (0.278 mg/g) was lower than that in *L. chinense* (0.360 mg/g). The total flavonoid content was found to be at least 3.2 times higher in *L. chinense* than that in ‘Ningqi No. 1’. On the contrary, a higher amount of isoflavone was observed in ‘Ningqi No. 1’, which increased by approximately 86% compared to *L. chinense* (Fig. 12).

## Discussion and conclusions

Wolfberry, a traditional medicinal herb and food supplement, contains a high concentration of bioactive constituents such as LBPs, carotenoids, betaines, flavonoids and vitamins [23]. Wolfberries have been reported to have significant antiaging effects, to enrich the liver and kidneys, and to participate in immune regulation and antitumour processes [1, 5]. In recent years, RNA-Seq has become an effective tool for studying gene expression in depth and detecting novel transcripts. In this study, 8817 DEGs were obtained during the fruit maturity stage of ‘Ningqi No. 1’ and *L. chinense* via Illumina sequencing technology. Numerous transcripts encoding putative genes involved in bioactive ingredient metabolism were explored using annotation. To gain a better understanding of these genes’ specific functions, in combination with the annotation information from the NR database and the PubMed database, 36 DEGs related to bioactive ingredient metabolism were identified that participated in carotenoid, flavonoid, terpenoid, alkaloid, vitamin metabolic pathways, etc. Interestingly, three transcription factors, *LbAPL*, *LbPHL11* and *LbKAN4*, were differentially expressed in this study*.*

**Fig. 13:**
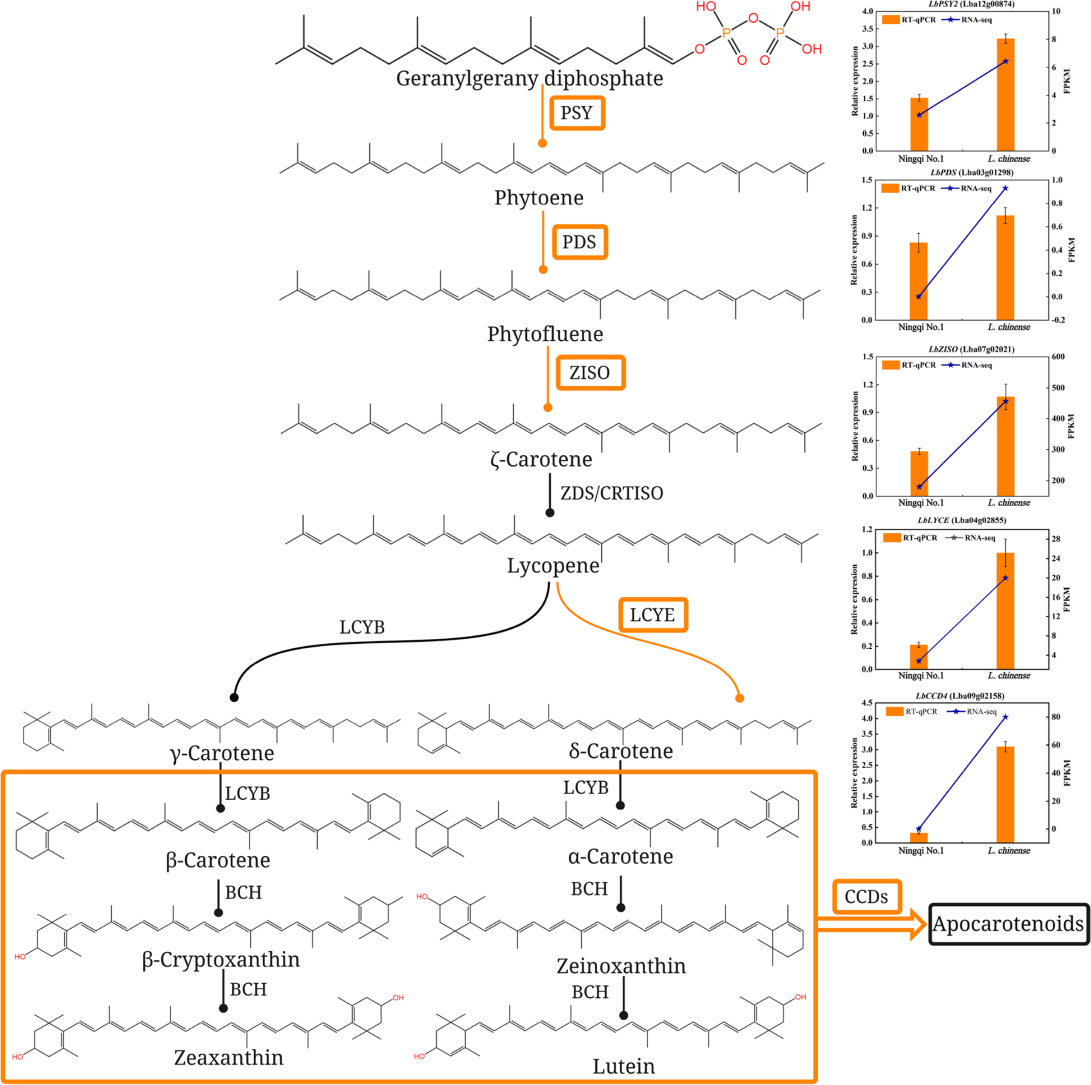
Schematic representation of the carotenoid biosynthesis pathway coupled with expression levels determined by RNA-seq and RT-qPCR analyses for each DEG. The enzyme names are abbreviated as follows: PSY, phytoene synthase; PDS, phytoene desaturase; ZISO, ζ-carotene isomerase; ZDS, ζ-carotene desaturase; CRTISO, carotenoid isomerase; LCYE, lycopene ε-cyclase; LCYB, lycopene β-cyclase; BCH, β-carotene hydroxylase; CCDs, carotenoid cleavage dioxygenases

Carotenoids, which are fat-soluble compounds, are the second largest group of metabolites of wolfberry. They are primarily composed of zeaxanthin (83%), β-cryptoxanthin (7%), mutatoxanthin (1.4%), β-carotene (0.9%), and other nutrients [8], which are essential for human health. In the present research, the transcriptome dataset contained 5 DEGs encoding related enzymes in the carotenoid biosynthesis pathway, including phytoene synthase 2 (*PSY2*), phytoene desaturase (*PDS*), 15-cis-zeta-carotene isomerase (*Z-ISO*), lycopene ε-cyclase (*LCYE*) and carotenoid cleavage dioxygenase 4 (*CCD4*). The important functions of these enzymes in carotenoid biosynthesis are as follows: the first step in the plant carotenoid biosynthesis pathway is to form phytoene, a two-step condensation reaction catalysed by *PSY* involving two GGPP molecules. Then *PDS* performs a two-step desaturation reaction of phytoene, converting it into phytofluene and ζ-carotene. The role of *Z-ISO* in transforming 9,15,9'-tri-cis-ζ-carotene to 9,9'-di-cis-ζ-carotene is critical to carotenogenesis in the darkness. In carotenoid biosynthesis, the third step consists of lycopene cyclization, in which *LCYE* attaches an ε ring to lycopene and forms δ-carotene. Most notably, these five genes were upregulated in *L. chinense* compared with ‘Ningqi No. 1’ (Fig. 13). Presumably, the increased expression of *PSY2*, *PDS*, *Z-ISO* and *LCYE* resulted in higher levels of α-carotene, α-cryptoxanthin, lutein, β-carotene, β-cryptoxanthin and zeaxanthin in *L. chinense* than in ‘Ningqi No. 1’, which led to the significantly higher total carotenoid content in *L. chinense* than that of ‘Ningqi No. 1’. However, downregulation of the carotenoid cleavage dioxygenase 4 (*StCCD4*) transcript level in transgenic potato has been shown to result in higher accumulation of violaxanthin and lutein than in the wild type, thereby increasing carotenoid levels [24]. In sweet *Osmanthus*, carotenoid cleavage dioxygenase 4 (*OfCCD4*) can cleave carotenoids such as β-carotene and zeaxanthin [25]. In addition, *CitCCD4*, which is found in citrus, can cleave β-cryptoxanthin and zeaxanthin [26, 27]. According to these reports, *CCD4* is a key gene that negatively regulates carotenoid accumulation. Intriguingly, *CCD4* was significantly upregulated in *L. chinense* in this study, which could enable the cleavages of β-carotene, β-cryptoxanthin, zeaxanthin and other carotenoids. Consequently, the content of total carotenoids would be reduced in *L. chinense*. However, since the identified five genes, including four genes that positively regulate carotenoid biosynthesis and only one negatively, all upregulated in *L. chinense*, the total carotenoid content in *L. chinense* could be still higher than that in Ningqi No.1. This hypothesis was consistent with the findings of the present study that the amounts of total carotenoids (0.360 mg/g) in *L. chinense* was approximately1.29 times higher in ‘Ningqi No. 1’ (0.278 mg/g). Obviously, *PSY2*, *PDS*, *Z-ISO*, *LCYE* and *CCD4* were suggested as good candidate genes for carotenoid biosynthesis in wolfberry, which needs to be further verified. In general, the identification of these crucial genes in the carotenoid biosynthesis pathway may help to elucidate the mechanism of carotenoid biosynthesis in ‘Ningqi No. 1’ and *L. chinense*. It could be of great importance to cultivate elite wolfberry species with high carotenoid content for reinforcing nutritional performances.

**Fig. 14:**
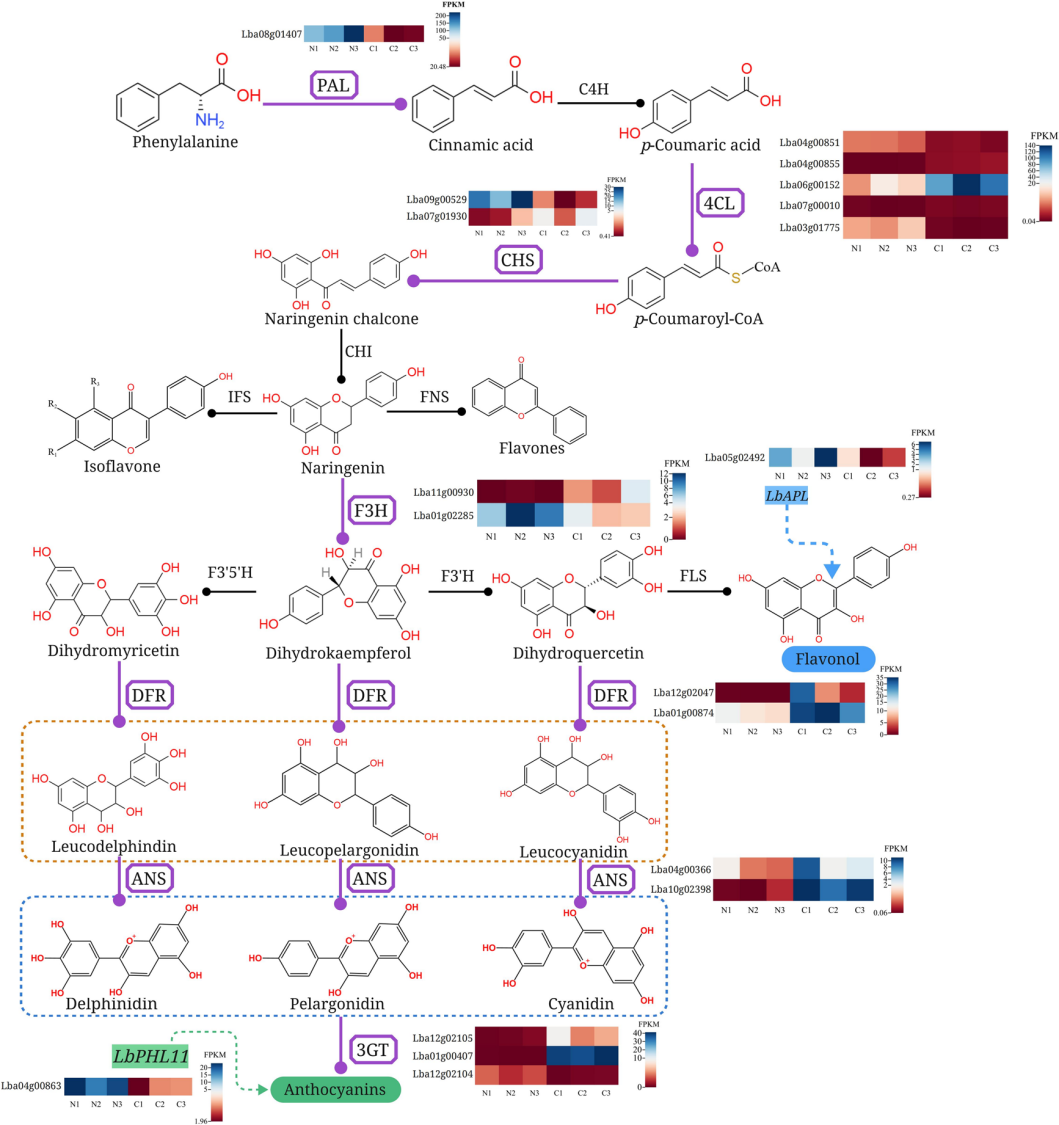
Schematic representation of the flavonoid biosynthesis pathway coupled with heatmaps of relevant genes involved in the pathway. The enzyme names are abbreviated as follows: PAL, phenylalanine ammonia lyase; C4H, cinnamic acid 4-hydroxylase; 4CL, 4-coumarate: CoA ligase; CHS, chalcone synthase; CHI, chalcone isomerase; FNS, flavone synthase; IFS, isoflavone synthase; F3H, flavanone 3-hydroxylase; F3′5’H, flavanone 3’,5’-hydroxylase; F3’H, flavonoid 3’-hydroxylase; FLS, flavonol synthase; DFR, dihydroflavonol 4-reductase; ANS, anthocyanidin synthase; 3GT, anthocyanidin 3-O-glucosyltransferase

The major component of goji berries are flavonoids, which have beneficial biological and pharmacological properties. Purified flavonoids extracted from goji berries, for example, dramatically improve the expression of six antiaging genes and play a pivotal role in ageing-related Alzheimer’s disease [28]. The present study revealed that a total of seventeen DEGs encoding seven enzymes, including one *PAL*, five *4CL*, two *CHS*, two *F3H*, two *DFR*, two *ANS* and three *3GT,* were involved in flavonoid biosynthesis (Fig. 14). Six downregulated and eleven upregulated unigenes were found among the seventeen DEGs. Recently, an abundance of experimental data has shown that transcriptional regulation affects active ingredient biosynthesis [29]. In particular, MYB transcription factors were closely relevant to control phenylpropanoid metabolism. For instance, 13 full-length cDNA clones of R_2_R_3_-MYB transcription factor (TFs) from *E. sagittatum* (EsMYB) have been isolated and characterized and were found to regulate the flavonoid biosynthetic pathway [30]. Moreover, it has been demonstrated that *GmMYB176* was in connection with flavonoid biosynthesis in soybean [31]. In this study, we found 3 MYB family transcription factors, APL (LbAPL), PHL11 isoform X2 (LbPHL11) and transcription factor KAN4 (LbKAN4), all downregulated in *L. chinense*. Significantly, *MYB27*, which is closely homologous to *LbPHL11*, is an anthocyanin repressor [21]. *LbPHL11* could thus play a critical role in anthocyanin biosynthesis in wolfberry. As is known, red wolfberry contains a small amount of anthocyanins. Precisely these identified DEGs can preliminarily explain the lower anthocyanin accumulation in ‘Ningqi No. 1’ and *L. chinense*. In this study, these six downregulated genes, including one *PAL*, two *CHS*, one *4CL*, one *F3H* and one *3GT,* were identified as potentially important in inhibiting anthocyanin accumulation in *L. chinense*. Additionally, the other eleven upregulated genes and the downregulated transcription factor *LbPHL11* may play a role in inhibiting anthocyanin biosynthesis in ‘Ningqi No. 1’. Of course, the flavonoid pathway is a complex process in which multiple enzyme metabolites are produced, including anthocyanin. Since they shared the same upstream pathway, these seventeen DEGs were also involved in other metabolic pathways, such as those of flavones, isoflavones and flavonols. In this study, the isoflavone contents in *L. chinense* were lower compared with ‘Ningqi No. 1’. Nevertheless, the total flavonoid levels detected in *L. chinense* were significantly higher than that of ‘Ningqi No. 1’, which might have been caused by the differential expression of pivotal enzyme genes in the flavonoid pathway, such as *PAL*, *4CL*, *CHS*. Notably, since *PAL* encoded by Lba08g01407 was the first enzyme in the phenylpropanoid biosynthesis pathway, the expression level of *PAL* was thus the basis of flavonoid biosynthesis. Furthermore, *PAL* expression levels were considerably higher in ‘Ningqi No. 1’ than in *L. chinense*, making it an important candidate gene for studying the difference in flavonoid and isoflavone content in the two types of wolfberries. Interestingly, the *LbAPL* identified in this study shared high ortholog with *MYB48*, and *MYB48* was found to regulate the process of flavonol biosynthesis in previous studies [22], which suggests that *LbAPL* could be a key transcription factor regulating flavonol content in wolfberry. Of course, future research is needed to confirm the role of these transcription factor genes in flavonoid biosynthesis.

In addition to carotenoids and flavonoids, wolfberry fruits are also rich in various vitamins, terpenoids and alkaloids. Among them, vitamins have numerous functions, such as scavenging free radicals, promoting hepatocyte proliferation, enhancing nonspecific immunity, and improving disease resistance [8, 32]. In addition, it is well known that many terpenoids are effective components of Chinese herbal medicine; they have been identified along with terpenoid glycosides in wolfberry. As one of the primary bioactive ingredients of wolfberry, alkaloids possess antioxidant properties and liver protection, neuroprotection, and antitumour properties [33–35]. In our research, four, four and five DEGs involved in vitamin, terpenoid and alkaloid metabolism pathways were identified, respectively. Three of the five DEGs involved in the alkaloid metabolic pathway participate in indole alkaloid biosynthesis, while the other two are involved in quinoline alkaloid biosynthesis. These results can provide basic data to elucidate the biosynthetic pathway and to conduct a dynamic analysis of the vitamin, terpenoid and alkaloid content in wolfberry.

To our knowledge, traditional breeding goals primarily focus on cultivating properties such as resistance to insect attack, early or late maturation, and increased yield. Enhancing functional and nutritional values is becoming a new trend in breeding objectives as people’s living standards improve. David Chagné et al. [36], for instance, demonstrated that the candidate genes *Leucoanthocyanidin reductase (LAR1)* and *hydroxy cinnamate/quinate transferase (HCT/HQT)* are likely to increase the content of polyphenolic compounds in apples, potentially facilitating the development of new apple cultivars containing fruits with higher concentrations of a variety of polyphenolic compounds with human health benefits. Furthermore, using de novo transcriptome assembly, researchers investigated the pivotal enzyme genes in the biosynthesis of chlorogenic acid in Korla fragrant pear, providing a theoretical basis for pear species selection and breeding in Xinjiang [11]. Therefore, this study focused on explaining the molecular basis of the difference between the bioactive components in ‘Ningqi No. 1’ and *L. chinense* fruits, identifying key genes and providing a wealth of transcriptome data as guidance for functional component research, future breeding of excellent strains and quality improvement in wolfberry.

## Materials and methods

### Plant materials and growth conditions

The major *L. barbarum* cultivar ‘Ningqi No. 1’ [37] and *L. chinense* [38] were cultivated in the standardization management field of the Qixin Wolfberry Seedling Professional Cooperatives in Zhongning, Ningxia Hui Autonomous Region, China (37°53′N, 105°72′E). Using the diagonal sampling method, the fruits of ‘Ningqi No. 1’ and *L. chinense* were harvested at the maturity stage, when the fruits were 1–2 times larger and bright red in colour compared with those at the green fruit stage (Fig. 1). Three biological replicates were collected, each of which included wolfberry samples from at least three diverse plants. The collected samples were grouped into two parts: one part was used for biochemical validation, and the other part was frozen at -80 °C for transcriptome sequencing analysis.

### RNA extraction and Illumina sequencing

Total RNA and mRNA extraction for sequencing was performed using Metwell Biotechnology Co., Ltd. (Wuhan, China). To ensure that the RNA met the quality requirements for constructing a sequencing library, the RNA purity, concentration and integrity were measured using the NanoPhotometer® spectrophotometer (IMPLEN, CA, USA), the Qubit® RNA Assay Kit in Qubit®2.0 Fluorometer (Life Technologies, CA, USA) and the RNA Nano 6000 Assay Kit of the Bioanalyzer 2100 System (Agilent Technologies, CA, USA). The cDNA library was constructed with the cDNA library construction kit (Beijing Genomics Institute, Shenzhen, China) and then sequenced using the Illumina NovaSeq 6000 platform at Wuhan Metwell Biotechnology Co., Ltd.

### Transcriptome data processing and de novo assembly

The raw sequencing image data was converted into raw reads by CASAVA base recognition. To get high-quality data, adapter sequences, empty reads, and low-quality reads with unknown base pairs ‘N’ greater than 10% and reads Q <  = 20 were eliminated from the raw paired-end reads through fastp [39], and then calculated the GC content of the clean reads. The Q20 and Q30 values were also generated by FastQC to evaluate the base quality. Then, HISAT2 2.1.0 software was utilized to align the clean read sequences from each sample to the *Lycium barbarum* reference genome [40], obtaining information about the location of the reference genome or genes as well as the unique sequence features of the sequencing sample [41]. StringTie version 1.3.4d software was used to de novo assemble the RNA-seq data into a transcriptome [42].

### Sequence annotation and classification

New transcript information was extracted from the comparison results of spliced transcripts and genome annotation, and then the sequence of new genes was collected from the genome. Using BlastX, the sequences were explored for annotation against the NCBI nonredundant (NR, http://www.ncbi.nlm.nih.gov) protein database with a cut-off E-value of 10^–5^. Blast2GO (version: 2.5.0, parameters: default) was used to retrieve Gene Ontology (GO) terms from the annotation of high scoring BLAST matches against the NCBI NR protein database (E-value ≤ 1.0 × 10^–5^, http://www.ncbi.nlm.nih.gov). Then, the GO categories were sorted with in-house Perl scripts. The Kyoto Encyclopedia of Genes and Genomes pathways (KEGG, http://www.genome.jp/kegg) were annotated against the KEGG database using Blastall software (version: 2.2.23, parameters: default). In addition, the sequences were annotated by aligning them in the Cluster of Orthologous Groups of proteins (KOG, https://www.ncbi.nlm.nih.gov/COG/), and the manual annotation and reviewing of protein sequences were conducted with the Swiss-Prot (http://www.expasy.ch/sprot) and Protein family databases (Pfam, https://www.ebi.ac.uk/interpro/entry/pfam/). The Plant Transcription Factor Database (PlantTFDB) and Pln TFDB were used to annotate and classify the transcription factors (TFs) using iTAK (v1.7 a) [43–45].

### Analysis of differentially expressed genes

Using ‘Ningqi No. 1’ fruits as a control, the transcriptome data from *L. chinense* fruits were analysed using high-throughput sequencing technology. The number of reads per gene was counted based on alignment results and information about the location of the gene on the *Lycium barbarum* reference genome. Fragments per kilobase of transcript per million fragments mapped (FPKM) of each gene were calculated as an index of gene expression level according to gene length and read count mapped to the gene. DESeq2 1.22.1 software was used to analyse the DEGs between the two groups [46, 47]. The false discovery rate (FDR) was calculated using *p* values that had been adjusted via the Benjamini‒Hochberg method. The DEGs were screened using |log2Fold Change|≥ 1.5 and FDR < 0.05. Then, GO function enrichment, KOG annotation and KEGG pathway enrichment of the DEGs were analysed.

### Real-time quantitative RT‒PCR (RT‒qPCR) analysis

Real-time quantitative PCR (RT‒qPCR) was utilized to verify the reliability of the RNA-seq results. Five key candidate genes were chosen for their important roles in carotenoid metabolism. The same RNA samples used in the transcriptome analysis were reverse transcribed with a HiScript® IIQ RT SuperMix for qPCR (+ gDNA wiper) kit (Vazyme Biotech Co. Ltd, Nanjing, China). Primer Premier 5.0 software was used to design the primer that was synthesized by Shanghai Sangong Biotechnology Co., LTD. The primers are listed in Table 4. The constitutively expressed gene *actin* (GenBank: HQ415754.1) from wolfberry was applied to normalize gene expression as an internal control. Real-time PCR was preformed in a 20 μL reaction mixture containing 50 ng of template cDNA, 10 μL of 2 × Universal SYBR Green Fast qPCR Mix (ABclonal Technology Co., Ltd, Wuhan, China), 0.4 μL of each primer and ddH_2_O. qPCR amplification was carried out on the qTOWER Real-time PCR Instrument (Analytik Jena AG, Germany). The following thermal cycling conditions were used: 95 °C for 3 min and 40 cycles of 95 °C for 5 s and 60 °C for 30 s. The 2^−ΔΔCt^ method was used to calculate the relative expression of DEGs [48]. We performed 3 independent bioreplicates per sample and 3 technical replicates per bioreplicate to ensure reproducibility and reliability of the experiment.

**Table 4:** Primers for real-time fluorescence quantitative PCR

Gene	Forward primer sequence (5’ → 3’)	Reverse primer sequence (5’ → 3’)
*LbPSY2*	TGAAGGAATGCGTATGGACTTGTGG	TCTCTGTCGTTGCCTTTGATTCAGG
*LbPDS*	AAAGGTTGTCGTGGAGCATCTAAC	ATAAGGTTGGTGTCTCAGCAATGTC
*LbLYCE*	GCGAGGGTGACATTGTGATTCC	ACCTCAACCTCCACTCCATAAGC
*LbCCD4*	TCCTCCTCGTCATCCTTCCATTG	TTCCTTTTACCACCTCGCATTCTG
*LbZISO*	CCGATATGATGGAGTGCAGTTATGG	CTTGTCAACAGCCGCTACTTCC
*LbKAN4*	CTCCTCCTACTCTTCCTGCTTCTC	GATGCTGGGACTGAAAAGGGTTC
*LbPHL11*	TGGTGGACCTGATAGCCTTGC	ATCTTGGTTCTTTGCCTGCTGTC
*LbAPL*	CACAGGAAACACTGGCAGGATATG	GCTGACAATGAAGAACTTGGACAAC
*actin*	CTTCCAGCCATCCATGATCGGTATG	AGCCACCACTGAGCACAATGTTAC

### Determination of carotenoid, flavonoid, and isoflavone content in the fruit maturity stage of the two wolfberry types

The determination of total carotenoid content was executed in accordance with a previously described method [49], with slight adjustments. The ground samples of 0.1 g wolfberry (triplicate) were extracted via ultrasound in 4 mL extraction solution (petroleum ether: acetone = 4:1) under dark conditions for 15 min at ambient temperature. Afterwards, the extracting solution was filtered and standardized to 4 mL through extraction solution, subsequently, extract was added into a 96—well plate. Finally, the absorbance values were recorded at 450 nm wavelength by a microplate reader (SpectraMax ABS plus, Molecular Devices, CA, USA), and carotenoid content was computed.

The NaNO_2_-AlCl_3_-NaOH approach based on the biochemical kit (NMKD0120, Norminkoda Biotechnology Co., Ltd. Wuhan, China) was utilized to determine the total flavonoid [50, 51]. The tissue homogenate of approximately 0.1 g wolfberry was added into 1.5 ml extracting solution, and then ultrasonically extracted in a water bath at 60℃ for 30 min. The suspension was centrifuged at 10,000 × g for 10 min at 25℃ to obtain an aliquot of extract. Subsequently, 1 mL of supernatant was placed in a 10 mL flask and combined with 4 mL deionized water, 0.3 mL NaNO_2_ (5%), and 0.3 mL AlCl_3_·6H_2_O (10%). The mixture was incubated at ambient temperature for 5–6 min followed by the utilization of 1 N NaOH. The solution was then brought up to 10 mL with 80% methanol, and its absorbance was measured at 510 nm wavelength using a spectrophotometer microplate reader. The total flavonoid content was expressed in mg/g FW.

An assay kit (NMKD0121, Norminkoda Biotechnology Co., Ltd. Wuhan, China) was utilized to measure the total isoflavone content at a 260 nm wavelength using a microplate reader (SpectraMax ABS plus, Molecular Devices, CA, USA) [52].

## Data Availability

The transcriptome raw reads have been deposited as a BioProject under accession: PRJNA962102 (https://dataview.ncbi.nlm.nih.gov/object/PRJNA962102?reviewer=b1lrilirtu04fvjfmjk4ng9nio). The materials are available from the corresponding author on reasonable request after the publication of the work.
